# Impact of aging on crossmodal attention switching

**DOI:** 10.1007/s00426-024-01992-3

**Published:** 2024-06-26

**Authors:** Ludivine A. P. Schils, Iring Koch, Pi-Chun Huang, Shulan Hsieh, Denise N. Stephan

**Affiliations:** 1 Institute of Psychology, RWTH Aachen University, Jägerstraße 17-19, 52066 Aachen, Germany; 2 Department of Psychology, National Cheng Kung University, University Road 01, Tainan, 701401, Taiwan

## Abstract

Previous studies on crossmodal visual-auditory attention switching using a spatial discrimination task showed performance costs when the target modality changed relative to when it repeated. The present study (*n* = 42 for each age group) examined age-related changes in crossmodal attention switching by asking young (age range 19 to 30 years old) and older (age range 64 to 80 years old) participants to respond to unimodal central cues and bimodal lateralized stimuli. The participants’ task was to indicate the location of the target in the relevant modality using button presses. Results showed general attention switch costs. Additionally, we found no specific age-related increase of attention switch costs (no difference in performance between switch and repetition of target modality), but age-related increased mixing costs (decreased performance for repetition in modality-mixed condition compared to single target modality). Moreover, spatial distraction produced a crossmodal congruency effect, which was only slightly larger in older adults. Taken together, age-related increased mixing costs suggest a general difficulty with maintaining more than one task, but no specific age-related crossmodal impairment in crossmodal attention switching.

## Significance

Public significance statement: Using a cued crossmodal visual-auditory spatial attention switching paradigm, our study provides evidence that older adults suffer from general slowing and from difficulty when performing multiple tasks at the same time. However, the ability to deal with crossmodal distraction does not seem to be impaired in older adults.

## Introduction

In everyday life, we have to selectively attend to stimuli in certain modalities while ignoring distractors in the same or different modalities. For example, while driving, we need to pay attention to road signs and ignore distracting noise such as cars passing by; but if we hear a horn, we have to instantly direct our attention towards the source. Processing these situations can be highly demanding, especially for older adults (Wasylyshyn et al., 2011). It is thus of high relevance to investigate at which point performance in such situations is influenced by aging. Studies already showed that attention switching can be influenced by aging (e.g., Karbach & Kray, 2009), by the modalities of a stimulus’ target and distractor (Evans & Treisman, 2010), and by how stimuli are displayed in the spatial environment (Longman et al., 2016). Our ability to switch between different modalities and tasks is constantly required.

In attention switching, participants need to flexibly switch attention between stimuli and tasks. The task-switching paradigm has been borrowed to investigate these processes (Rogers & Monsell, 1995). This paradigm usually comprises single-task blocks (only one type of task is performed in alternation) and mixed-tasks blocks (two or more different tasks are performed), allowing one to calculate two different measures: mixing costs (performance difference in single-task trials and repetition trials in mixed-tasks blocks) and switch costs (performance difference between repetition trials and switch trials within mixed-tasks blocks). Mixing costs represent the cognitive load (i.e., the working memory load) needed to hold multiple tasks in memory simultaneously and update them (Kray & Lindenberger, 2000), whereas switch costs reflect the ability to flexibly switch from one task to another by activating the currently relevant task while inhibiting the currently irrelevant task (see, e.g., Koch et al., 2018, for a review). Interestingly, selective attention switching has demonstrated to be directly impacted by cognitive load (Lavie et al., 2004) and flexibility (Koch et al., 2018), with increased interference of distractors when participants need to switch between tasks.

Research showed that aging is accompanied by a general slowing in response time (RT), but it also suggested a decline in some specific cognitive processes, such as in inhibition, working memory, and flexibility (Salthouse, 1996; Verhaeghen & Cerella, 2002). For example, the inhibitory deficit hypothesis states that older adults tend to be impaired in ignoring distractors (Hasher et al., 2007; Hasher & Zacks, 1988). Most importantly, some studies showed that older adults are impaired when switching between tasks. However, once the general reduction of processing speed is taken into account, studies often demonstrate age-equivalent switch costs (see Wasylyshyn et al., 2011, for a meta-analysis). This includes performance in attention switching settings (Lawo & Koch, 2014). In contrast, the age-related increase of mixing costs was more consistent (e.g., Kray & Lindenberger, 2000; Lawo et al., 2012; see also Wasylyshyn et al., 2011), implying age-related difficulties with monitoring and updating attentional sets.

At this point, it is relevant to define the concept of *task* as we understand it in the context of our study. A widely recognized conception is that each task requires the activation of a “task-set”. Monsell (2003) described a task-set as the mental representation of task goals (i.e., the representation of the set of stimuli, such as cues, targets and distractors) and their corresponding rules (i.e., which response matches to which target). In most task-switching experiments (e.g., Hirsch et al., 2016; Meiran, 1996; Rogers & Monsell, 1995), the rules generally differ from task to task so that each response can be associated with more than one rule. However, this is not necessary to distinguish between two tasks: as soon as one component of the task-set differs, they can be categorized as distinctive tasks. For example, in spatial tasks participants have to indicate on which side a stimulus is presented. With bimodal stimuli (auditory and visual at the same time, i.e., bivalent stimuli), stimuli in each modality can be presented unilaterally on the left or on the right in each trial (i.e., a combination of a target-modality and a distractor-modality), so that a shift in target modality can be considered a task shift. In mixed-tasks, the relevant modality varies from trial to trial, along with the task (or task-set), although the actual spatial response rule (i.e., “press left when target modality is on the left”, for both modalities) remains the same regardless of the target modality.

Both mixing and switch costs are modulated by the modalities of the cues and the stimuli (Kreutzfeldt et al., 2016; Lukas et al., 2010b). For example, modality switch costs were observed when participants needed to switch between modalities from trial to trial, compared to when they could attend to the same modality twice in a row (Lukas et al., 2010b). Many studies highlight an age-related impairment in inhibiting certain modalities, stressing how crucial the role of modalities is in age-related distraction (Chen & Hsieh, 2023; Juncos-Rabadàn et al., 2008; Pick & Proctor, 1999). Generally, modality-related switch costs can be interpreted as an activation bias towards the relevant modality of a stimulus, with this bias requiring greater activation in switch trials than in repetition trials, thus requiring more time to orient our attention to the target stimulus in the switch trials.

Modality-related switch costs also have been observed in crossmodal task-switching when using bivalent stimuli, with the target and the distractors displayed in different modalities (bimodal stimuli; Lukas et al., 2010a, b). A well-known effect observed with bivalent stimuli is the congruency effect: performance is better when the distractor and the target lead to the same response (e.g., both requiring a right button press; congruent stimulus) than when they lead to different responses (e.g., the target modality requiring a right button press, but the distractor modality would require a left button press; incongruent stimulus). Interestingly, only a few studies investigated the impact of age on crossmodal (auditory and visual) distractor processing. Following a review by Guerreiro and colleagues (2010), some studies suggest that crossmodal distraction might be prone to age-related differences. Research on multisensory processing goes in the same direction (de Dieuleveult et al., 2017), although the focus of multisensory integration is towards sensory, pre-attentive processes, whereas crossmodal distraction focuses on target selection (i.e., attentional processes).

Implementing bimodal stimuli in a selective attention task-switching paradigm allows one to investigate whether cognitive load and task-set inhibition impact on crossmodal selective attention. In single-task blocks, only one modality needs to be attended during the block and the attentional weight (or load) is heavily biased towards that modality. Instead, in mixed-task blocks, the cues can indicate different target modalities from trial to trial, so that both modalities must remain attended to some degree, regardless of the current modality that the cue indicates. As a consequence, in repetition trials, the weight of the irrelevant modality will remain less downgraded compared to in single-task blocks. In contrast, in switch trials, the currently irrelevant modality was relevant in the preceding trials, leading to a higher activation, and thus a higher weight, of the irrelevant modality compared to repetition trials (Koch et al., 2018).

Most previous studies focused on unimodal distraction, and only few studies assessed age-related crossmodal distraction (Guerreiro et al., 2010). More precisely, no study ever examined age-related flexibility in crossmodal distraction. Although spatial tasks might be less susceptible to lead to age-related crossmodal distraction (see e.g., Guerreiro et al., 2014; Guerreiro et al., 2012), working memory tasks displayed the reverse pattern (Guerreiro & Van Gerven, 2011; Guerreiro et al., 2013; Van Gerven & Guerreiro, 2016). In addition, the capacity to inhibit distractors (as measured, for example, by the congruency effect) is negatively impacted as cognitive load increases (Lavie et al., 2004), as well as when participants need to switch from one task set to another (Koch et al., 2018). Accordingly, cognitive load capacities, but potentially not crossmodal spatial distraction, is negatively impacted by aging. Hence, we aim to investigate if working memory load increases crossmodal distraction similarly for older and young adults by implementing a crossmodal spatial selective attention task within a task-switching paradigm.

Taken together, previous studies did not yet investigate the impact of aging on crossmodal allocation of attention in attention switching. To fill this gap, we compared the performance of older and young adults in a crossmodal spatial attention switching task, using the task-switching methodology. Specifically, we employed unimodal (visual *or* auditory) cues and bimodal (visual and auditory) stimuli. The modality of the cue indicated the target modality. Cues were presented centrally and the stimuli were each presented visually and auditorily, on the left or right side, one being a target and the other the distractor (i.e., only crossmodal distraction). Participants indicated via button press on which side the target was presented. In single-task blocks, the cue indicated always the same target modality throughout the whole block of experimental trials while stimuli were bimodal. In mixed-task blocks, the cued modality randomly varied between visual and auditory from trial to trial.

First, we expected to find generally longer RT for older compared to young participants due to general slowing (Salthouse, 1996). We also expected larger mixing costs for older than young participants (age-related mixing costs), but not larger switch costs (age-equivalent switch costs; Wasylyshyn et al., 2011). Finally, as older adults tend to display larger crossmodal distraction in working memory tasks and larger distractor sensitivity with higher working memory load, we expected a larger congruency effect for older than for young adults in mixed-tasks blocks (Guerreiro & Van Gerven, 2011; Guerreiro et al., 2013; Van Gerven & Guerreiro, 2016; Lavie et al., 2004). Indeed, working memory load in our study is manipulated by increasing the number of task sets to be processed within blocks, which slightly deviates from the experiments cited in the above-mentioned studies (perceptual load, notably via identity-based distraction). Hence, our study additionally allows to examine whether age-related performance is influence by the *type* of cognitive load that is enforced on them.

## Method

### Participants

The final sample of this online experiment^1^ consisted in 42 older participants (15 males, mean age = 68 (± 3.7) years, age range 64–80) and 42 young participants (13 males, mean age = 24 (± 3.4) years, age range 19–30). Participants were recruited via Prolific (www.prolific.co)^2^. All had normal or corrected-to-normal vision and hearing, and no neurological or cognitive impairments. The use of a headset and hearing ability was assessed via a hearing test available on Gorilla (https://gorilla.sc). In addition, it was tested whether participants could indicate the location of a tone displayed on their left or right side correctly. This also ensured that the intensity of the auditory stimuli was sufficient. An adapted online version of the DemTect (Kalbe et al., 2004) was used to screen for early dementia^3^. Only participants who passed the hearing test and the DemTect took part in the study. They all gave their informed consent and received 10€ as compensation.

### Stimuli, tasks and responses

Participants had to indicate the spatial location of the target in the relevant modality, indicated by a cue in the same modality (i.e., modality compatible) by pressing the F (left) or J (right) key on their keyboard with the index finger of the respective hand. Visual stimuli were semi-transparent white diamonds displayed horizontally on the left or right side of the fixation cross^4^. Auditory stimuli were lateralised 600 Hz tones presented in the left or right ear. Visual cues consisted of four white asterisks horizontally aligned on both sides of the fixation cross. Auditory cues consisted of binaural 400 Hz tones. If an incorrect response was given, bimodal error feedback consisting of two red crosses and two short 200 Hz tones sawtooth (sharp and non-sinusoidal) waves was presented for 200 ms.

The stimulus-response (S-R) mapping was spatially compatible. During the single-task blocks, stimuli were bimodal, cues were consistently presented in the same modality, and participants had to indicate the location of the target in this modality only. In the mixed-tasks blocks, stimuli were also bimodal, but the cue modality varied randomly and participants had to respond to the target presented in the same modality as the cue.

### Procedure

Gorilla software controls which type of device is used (in our case only allowing laptop or PC) and ensures that a minimum requirement for the internet connection is met (minimum of 2 Mbps required). Preliminarily, participants were instructed to work undisturbed for one hour, in a calm and not too bright room. The experiment started after participants gave their informed consent, followed by passing the hearing assessment and the DemTect. During the hearing assessment, participants tested the stimuli that would be presented during the experiment and were instructed to adjust the loudness.

At first, participants filled out the demographic data form. Afterwards, written instructions and explanatory figures were displayed on the screen before each practice task and regular task. Participants were asked to keep looking at the fixation cross in the centre of the screen throughout the entire experiment.

The experiment began and ended with two single-task blocks of 80 trials each, framing eight mixed-tasks blocks of 80 trials each (performance for single-task blocks was calculated by computing all four blocks, in order to account for potential practice effect). The order of single-task blocks (visual vs. auditory targets) was counterbalanced across participants. The first two single-task blocks and the first two mixed-tasks blocks were preceded by two practice blocks of 16 trials. The order of the single-task blocks, the sequence of stimuli and cues, and the number of modality switches in mixed-tasks blocks were counterbalanced.

At the beginning of each task, participants were encouraged to respond as quickly and accurately as possible and were informed that they would be excluded from the experiment if they were making too many incorrect responses. Accordingly, a pre-screening criteria (also intended as an attentional check) was implemented: participants with more than 70%^5^ of incorrect responses (practice trials included) in any of the two first single-task blocks were excluded, leading to the exclusion of 12 older and 2 young participants.

**Fig. 1:**
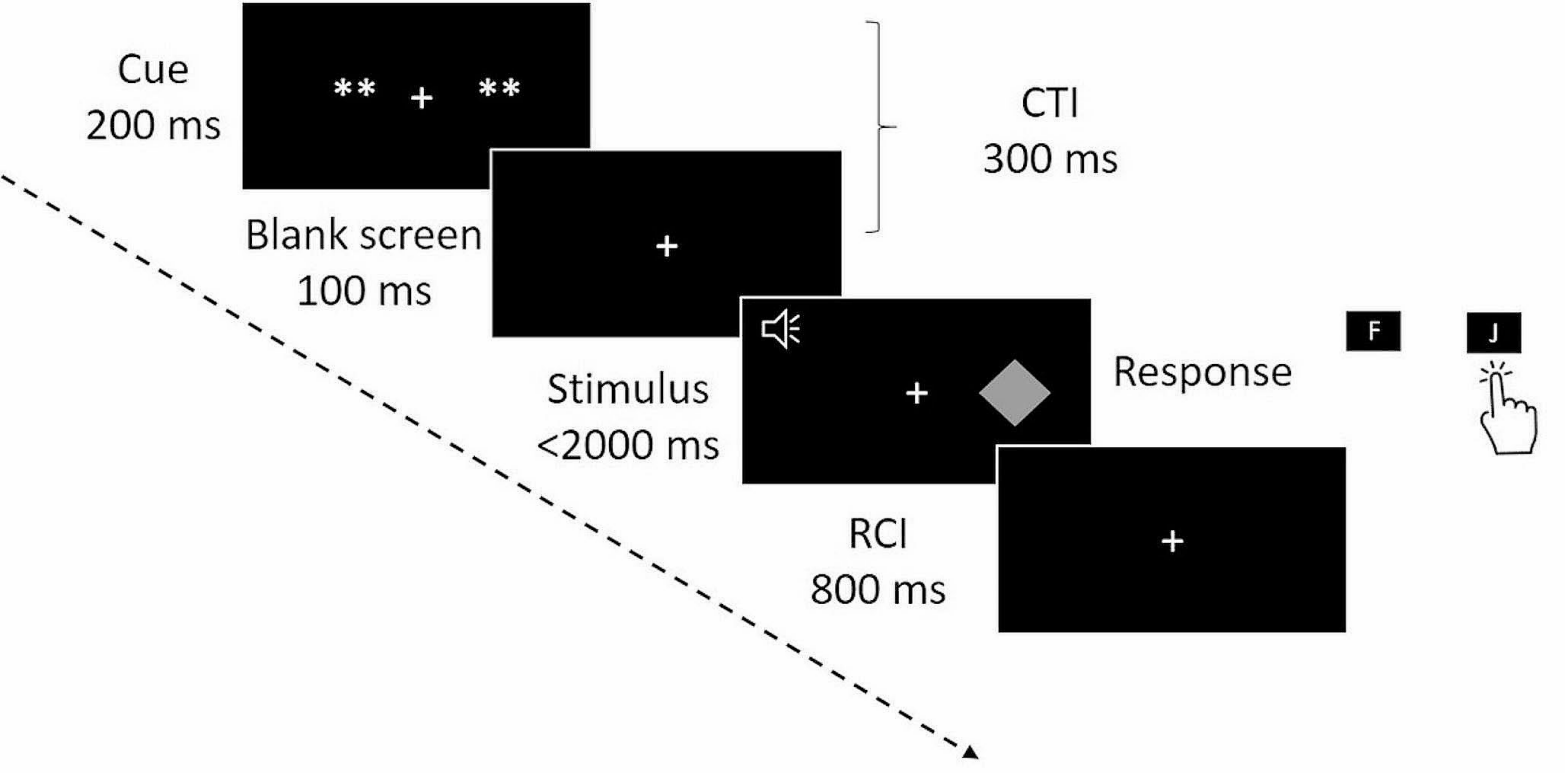
Timeline of a trial. *Note.* Example of a visual trial. The visual cue appears for 200 ms, followed by 100 ms of blank screen leading to a cue-target interval (CTI) of 300 ms in total. Then, the visual target stimulus and the auditory distractor stimulus appear until participant’s response (with a maximum of 2000 ms). The response is followed by 800 ms of response-cue interval (RCI)

Cues were displayed for 200 ms at the beginning of each trial, followed by 100 ms blank screen and silence so that the CTI was kept constant at 300 ms. The maximum response time was 2000 ms. In case of error or non-response, an error feedback was presented for 150 ms followed by 50 ms of blank screen and silence. The response-cue interval (RCI) was kept constant at 800 ms (1000 ms in case of error) and the total response-stimulus interval (RSI, i.e., RCI + CTI) was 1100 ms (lengthened to 1300 ms in case of error). Figure 1 depicts the timeline of a trial.

### Design

Mixing and switch costs were analyzed in separate contrasts. For the *mixing costs analysis*, independent within-subject variables were transition (repetition of target modality in mixed-tasks vs. single-task block) and congruency (incongruent vs. congruent). For the *switch costs analysis*, independent within-subject variables were transition (switch of target modality vs. repetition of target modality) and congruency (incongruent vs. congruent). (A vizualisation of how mixing costs and switch costs are obtained from the single and mixed task blocks is available in Koch & Kiesel, 2022; Fig. 1.) The independent between-subject variable was age (older vs. young) for both contrasts. RTs, and ER were the dependent variables. To account for general slowing in older adults, the dependent variables for effects including the variable age were log(RT) and ER. Indeed, information processing slows down with aging, and log transformation allows to rescale both age groups to a common scale (Kray & Lindenberger, 2000). This means that any age-related difference that remains after log transformation reflects differences in processes that are not disproportional, and not linked to the age-related diminution in processing speed (Faust et al., 1999; Salthouse, 1985). The significance threshold was fixed at an alpha level of .05.

## Results

### Data filtering

Incorrect spatial responses were considered as errors, and non-responses were considered as time-outs. Practice trials and the first trial of each block were excluded from the analysis. Afterwards, we calculated technical errors, that is, trials where the time-duration specified for each parameter (auditory and visual features of the different stimuli) could not be met by the hardware (0.8%). Data of participants with more than 15% of technical errors were excluded from the analysis (two older participants).

Then, we excluded the data of participants with less than 25% of correct responses in incongruent auditory or visual trials (one older, one young), because of the high chance that they did not manage to perform the task correctly. After this, we excluded trials with RTs below 100 ms (0.2%), and trials following an error (7.8%) in the data sets for all remaining participants. For each participant, mean and standard deviation were calculated. We excluded RTs of correct trials deviating more than 3SD from their individual condition mean (1.9%)^6^. Errors were discarded from the RT analysis (6.5%). Hence, in total, 85.3% of the total amount of trials were used for RT analysis, and 91.2% of the total amount of trials for ER analysis. Finally, in order to account for the general slowing of RT observed for older participants, a log transformation was applied on RT for interaction that included the age variable (Faust et al., 1999; Salthouse, 1996). Timeout trials (participants do not answer within the time allowed for stimulus presentation) were removed from the analysis of RT and ER and were not further investigated due to their low occurrence (0.1%).

### Mixing costs contrast

**Fig. 2:**
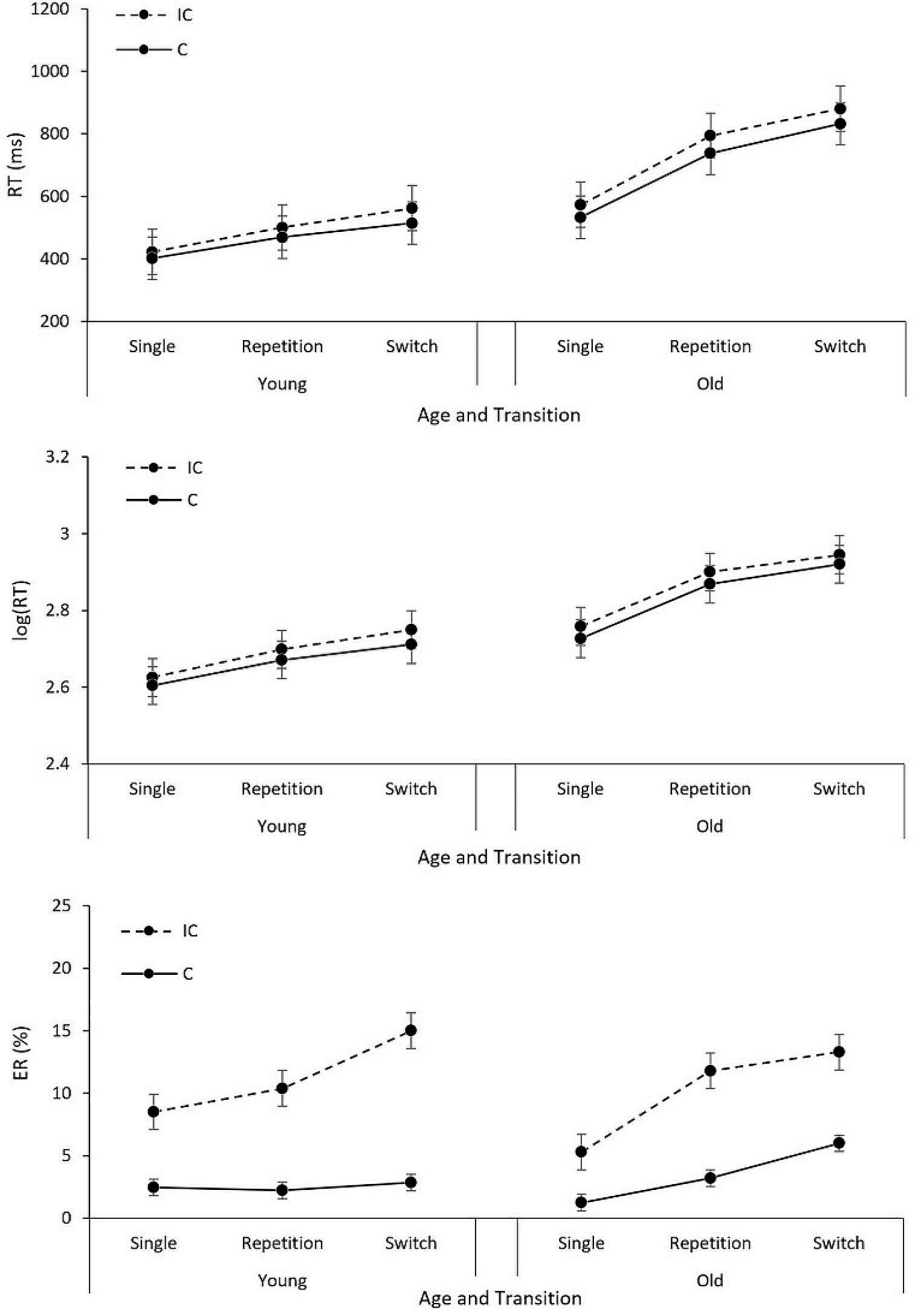
RT in ms (upper panel), log(RT) (middle panel), and ER in % (lower panel) as a function of congruency, transition, and age group. *Note.* IC = Incongruent trials, C = Congruent trials

For RT and ER, we ran an analysis of variance (ANOVA) with the independent variables age, congruency, and transition (single target modality vs. repetition of target modality). Descriptive information available in Table 1, and Fig. 2 provides an overview of these variables for RTs and ERs. Additionally, we reported the results with log-transformed RTs for significant effects of age-group, to account for the general slowing in older adults’ performance.

**Table 1:** RTs in ms (upper panel), log(RT) (middle panel) and ER in % (lower panel) for transition, age group and congruency

RT	Young			Older		
	IC	C	Cong Effect	IC	C	Cong Effect
Switch	562	514	48	880	832	48
Repetition	500	469	31	794	738	56
Single	422	402	20	573	533	40
Switch costs	62	45		86	94	
Mixing costs	78	67		221	205	
log(RT)	Young			Older		
	IC	C	Cong Effect	IC	C	Cong Effect
Switch	2.75	2.71	0.04	2.94	2.92	0.02
Repetition	2.70	2.67	0.03	2.90	2.87	0.03
Single	2.63	2.60	0.02	2.76	2.73	0.03
Switch costs	0.05	0.04		0.04	0.05	
Mixing costs	0.07	0.07		0.14	0.14	
ER	Young			Older		
	IC	C	Cong Effect	IC	C	Cong Effect
Switch	15.0	2.9	12.1	13.3	6.0	7.3
Repetition	10.4	2.3	8.2	11.8	3.2	8.6
Single	8.5	2.5	6.0	5.3	1.3	4.1
Switch costs	4.6	0.6		1.5	2.8	
Mixing costs	1.9	-0.2		6.5	2.0	

*Note.* IC = Incongruent trials, C = Congruent trials, Cong Effect = Congruency effect

### RT

For RT, there was a main effect of age, *F*(1,82) = 122.40, *p <* .001, η_p_^2^ = 0.599, with longer RTs for older (660 ms) than younger (448 ms) adults. This effect was confirmed by the log RT analysis, *F*(1,82) = 143.92, *p* < .001, η_p_^2^ = 0.637. The main effect of congruency was also significant, *F*(1,82) = 142.98, *p* < .001, η_p_^2^ = 0.636, indicating longer RTs for incongruent (573 ms) than congruent (536 ms) trials. The interaction of age and congruency was significant, too, *F*(1,82) = 12.95, *p* < .001, η_p_^2^ = 0.136, but was not confirmed by the log RT analysis, *F*(1,82) = 1.70, *p* = .196, η_p_^2^ = 0.020.

The main effect of transition was significant, *F*(1,82) = 192.51, *p* < .001, η_p_^2^ = 0.701, indicating longer RTs for repetition (625 ms) than single (483 ms) trials. As expected, the interaction of transition and age was also significant, *F*(1,82) = 46.55, *p* < .001, η_p_^2^ = 0.362, indicating clearly larger mixing costs for older (213 ms) than young (73 ms) adults. This interaction was confirmed using log RT, *F*(1,82) = 23.68, *p* < .001, η_p_^2^ = 0.224. The interaction of transition and congruency was also significant, *F*(1,82) = 7.27, *p* = .009, η_p_^2^ = 0.009, indicating larger congruency effect for repetition (43 ms) than single (30 ms) trials. The three-way interaction of age, congruency and transition was not significant, *F*(1,82) = 0.32, *p* = .574, η_p_^2^ = 0.004.

### ER

In ER, we found no main effect of age, *F*(1,82) = 0.30, *p* = .586, η_p_^2^ = 0.004. The main effect of congruency was significant, *F*(1,82) = 190.14, *p* < .001, η_p_^2^ = 0.699, indicating larger ER for incongruent (9.0%) than congruent (2.3%) trials. The interaction of congruency and age was not significant, *F*(1,82) = 0.62, *p* = .432, η_p_^2^ = 0.008.

The main effect of transition was significant, *F*(1,82) = 31.07, *p* < .001, η_p_^2^ = 0.275, indicating larger ER for repetition (6.9%) than single (4.4%) trials. As expected, the interaction of age and transition was also significant, *F*(1,82) = 14.13, *p* < .001, η_p_^2^ = 0.147, indicating larger mixing costs for older (4.2%) than young (0.8%) adults. As in RT, the interaction of transition and congruency was significant, *F*(1,82) = 22.34, *p* < .001, η_p_^2^ = 0.214, indicating a larger congruency effect for repetition (8.4%) than single (5.1%) trials. The three-way interaction of transition, congruency, and age pointed towards a trend, *F*(1,82) = 2.96, *p* = .089, η_p_^2^ = 0.035. The trend suggests that older adults have a rather increased congruency effect in repetition (8.6%) compared to single (4.1%) trials, *t*(41) = 5.06, *p* < .001; compared to young adults (repetition trials = 8.2%, single trials = 6.0%, *t*(41) = 2.57, *p* = .018).

### Switch costs contrast

For RT and ER, we ran an analysis of variance (ANOVA) with the independent variables age, congruency, and transition. Descriptive information available in Table 1, and Fig. 2 provides an overview of these variables for RTs and ERs.

### RT

There was a main effect of age, *F*(1,82) = 159.27, *p* < .001, η_p_^2^ = 0.660, indicating larger RTs for older (811 ms) than young (511 ms) adults. This effect was confirmed using log RT, *F*(1,82) = 178.76, *p* < .001, η_p_^2^ = 0.686. There was a main effect of congruency, *F*(1,82) = 113.40, *p* < .001, η_p_^2^ = 0.580, showing longer RTs for incongruent (684 ms) than congruent (638 ms) trials. This effect was replicated using log RT, *F*(1,82) = 109.39, *p* < .001, η_p_^2^ = 0.572. The interaction of congruency and age was not significant, *F*(1,82) = 2.10, *p* = .151, η_p_^2^ = 0.025. The main effect of transition was significant, *F*(1,82) = 214.41, *p* < .001, η_p_^2^ = 0.723, showing longer RTs for switch (697 ms) than repetition (625 ms) trials. The interaction of transition and age was significant, *F*(1,82) = 13.97, *p* < .001, η_p_^2^ = 0.146, showing larger switch costs for older (90 ms) than young (56 ms) adults. However, this was not confirmed using log RT, *F*(1,82) = 0.35, *p* = .557, η_p_^2^ = 0.004. The interaction of transition and congruency was not significant, *F*(1,82) = 1.11, *p* = .296, η_p_^2^ = 0.013.

Interestingly, the three-way interaction of transition, congruency and age was significant, *F*(1,82) = 7.72, *p* = .007, η_p_^2^ = 0.086. This was confirmed using the log RT analysis, *F*(1,82) = 11.93, *p* = .001, η_p_^2^ = 0.127. Hence, the follow up analysis was conducted using log RT. Switch trials had a significantly smaller congruency effect for older (0.05) than young (0.09) adults, *t*(82) = 2.24, *p* = .028. However, repetition trials displayed no difference in the congruency effect between age-groups, *t*(82) = 0.515, *p* = .608.

### ER

There was a main effect of congruency, *F*(1,82) = 108.77, *p* < .001, η_p_^2^ = 0.570, showing higher ER for incongruent (12.6%) than congruent (3.6%) trials. The interaction of congruency and age was not significant *F*(1,82) = 1.60, *p* = .210, η_p_^2^ = 0.019.

The main effect of transition was significant, *F*(1,82) = 26.07, *p* < .001, η_p_^2^ = 0.241, showing higher ER for switch (9.3%) than repetition (6.9%) trials. There was no main effect of age, *F*(1,82) = 0.42, *p* = .519, η_p_^2^ = 0.005. Neither the interaction of transition and age, *F*(1,82) = 0.26, *p* = .615, η_p_^2^ = 0.003, nor the interaction of transition and congruency, *F*(1,82) = 2.54, *p* = .115, η_p_^2^ = 0.030, were significant.

Interestingly, the three-way interaction of transition, congruency and age was significant, *F*(1,82) = 9.95, *p* = .002, η_p_^2^ = 0.108. Switch trials had a significantly smaller congruency effect for older (7.3%) than young (12.1%) adults, *t*(82) = 2.06, *p* = .042. However, repetition trials did not display a significant difference in the congruency effect between older and young adults, *t*(82) = 0.314, *p* = .754.

## Discussion

In our study using task-switching methodology, we investigated how age impacts performance in crossmodal spatial attention switching. We compared young and older adults’ performance in a spatial localisation task involving bivalent (bimodal auditory-visual) stimuli and unimodal (auditory or visual) cues. Participants responded spatially compatibly to the target in the cued modality.

In the mixing cost and switch cost contrast, older adults displayed longer RTs, confirmed with the log(RT) analysis. In the switch-cost contrast, for both log(RT) and ER, the modulation of the switch costs by the congruency effect depended on age: switch trials displayed a smaller congruency effect for older than young adults, but repetition trials displayed similar congruency effects across age-groups. The mixing costs contrast additionally showed larger mixing costs for older adults.

Regarding age-related effects, we first found overall slower responses in older adults, consistent with general slowing as predicted by the processing speed account (Salthouse, 1996). Older adults also showed larger mixing costs, suggesting, as previously explained, problems with monitoring and updating attentional sets (Kray & Lindenberger, 2000). Switch costs were also increased for older adults, but this difference disappeared with log(RT), that is, when controlling for general slowing. This means that, although older adults do display larger switch costs, there appears to be no “real” age-related impairment in the shifting of attention itself (for a meta-analysis of age-related effects, see Wasylyshyn et al., 2011; see also Chen & Hsieh, 2023, for a recent meta-analysis).

Former studies showed that older adults tend to be better at using an alerting cue (similar as cues in single task blocks), but have more difficulties at modulating sensory processing in a top-down manner (similar as cues in mixed task blocks; Wiegand et al., 2017). This could have contributed to the larger mixing costs observed in older adults in our study, while remaining independent of modality processing (Lukas et al., 2010b; Wiegand et al., 2019). Additionally, it is interesting to note that some studies found age-related switch costs, contrary to ours. In line with the multiple components of task switching, we suggest that cognitive processes related to task switching can be decomposed in three phases, and that every modification in one of these phases might impact the size of the observed (age-related) switch costs (Ging-Jehli & Ratcliff, 2020). Although this means that switch costs will depend on many factors and that their (absence of) occurrence might be difficult to generalize, our study highlights that it is unlikely to observe age-related switch costs in crossmodal situations when the primary task is a location task, and that stimuli and stimuli-response mappings are easy to process (for example, taking note during a talk or looking for supplies in a supermarket).

In our crossmodal spatial attention switching paradigm, distractor processing and selective attention are indexed by the congruency effect. The congruency effect we found confirms that selective attention and distraction take place not only within but also across modalities. Older adults demonstrated a larger congruency effect, which goes in line with the larger congruency effect observed in multisensory situations (de Dieuleveult et al., 2017). However, this effect disappeared with the log transformation, suggesting that the difference in the congruency effect can rather be attributed to a general slowing in processing speed, and that there is no specific age-related crossmodal distraction.

In the mixing costs contrast analysis, data showed a larger congruency effect in repetition trials of mixed task blocks than in single task blocks. As expected, this suggests that selective attention is directly impacted by cognitive load (Lavie et al., 2004). We explain the increase of the congruency effect in the mixing costs contrast by assuming that it is more difficult to keep both visual and auditory modalities in a state that allows frequent shifts in attentional weight to modality, compared to the single-task blocks. However, this impact of modality mixing on the congruency effect was not modulated by age, so that the increased congruency effect in mixed blocks did not differ between older adults and young adults. This was the case despite older adults displaying larger mixing costs than young adults. Thus, our study emphasizes the limited impact of cognitive load on modulating the congruency effect (Kiesel et al., 2007) in spatial crossmodal situations.

In the switch cost contrast analysis, the congruency effect was larger for young adults than older adults, but only in switch trials (similar congruency effect across age-groups for repetition trials). This suggests that the additional attentional demands for older adults in our paradigm mostly arose already in the repetition trials of the mixed blocks. We propose that older adults might have a disproportionate difficulty to process the higher cognitive load induced in mixed tasks. Hence, they would show a specifically poor performance already in repetition trials, and this, independent of the level of distraction (high or low distraction, as indexed by the congruency effect). This decreases the congruency effect when comparing repetition and switch trials (i.e., distraction-related switch costs).

Therefore, we can conclude from our data that, while finding a number of relevant age-related performance differences in the mixing costs analysis, there does not appear to be a consistent age-related deficit in crossmodal selective attention. Indeed, previous studies showed that age-related impairment with crossmodal targets and distractors appears for example in verbal working memory tasks (Guerreiro & Van Gerven, 2011; Guerreiro et al., 2013). In comparison, previous studies that used spatial tasks with crossmodal targets and distractors showed equivalent performance across age groups (Guerreiro et al., 2012, 2014). Similarly, in the present context of crossmodal spatial attention switching, we likewise did not observe a larger crossmodal sensitivity to distraction for older adults. This means that if there are any age-related effects at all in crossmodal spatial attention switching, then they may be fairly small.

Limitations of this study, notably due to the online experimental setting, must also be specified. First, we might argue that older adults that performed the task were probably high-performing ones, as the online setting implies the use of a laptop and Prolific without experimenter’s instructions. Second, it is important to note that the volume could be individually adjusted by each participant, which could have lowered possible modality-related differences, for example because older adults might have turned up the volume of the auditory stimuli. However, Guerreiro and van Gerven (2011) also previously showed that performance in crossmodal auditory-visual distraction is probably not linked to hearing performance: in their crossmodal auditory-visual task, the correlation analysis demonstrated no relation between hearing performance and performance to the task.

In conclusion, in our study, we found increased mixing costs in older adults, suggesting generally more difficulties with task-set updating in older adults than in young adults in the present crossmodal spatial attention switching paradigm. However, in these crossmodal situations, older adults do not appear to have specific age-related crossmodal impairments. Together with previously published findings using spatial tasks without any attention switching requirement, our findings using an attention switching manipulation suggest that age-related crossmodal impairments might be less likely observed in spatial tasks. Further research is thus needed to clarify under which conditions age-related differences in crossmodal distraction might be observed in spatial tasks.

## Data Availability

Data and code used to analyze the data are available on OSF on the following address: https://tinyurl.com/ypwktaav.

## Footnotes

1 Minimum sample size was determined considering statistical power in a between-subject design. A large age-related effect was assumed (Cohen’s d = 0.80), as well as a statistical power of 0.9. Hence, at least 2*34 participants were required.

2 We pre-screened participants on Prolific, meaning that this experiment was available for around 18,600 young and 812 older adults on the platform. Additionally, participant’s nationality was distributed as follows: for young adults, 95.2% UK, 2.4% USA, 2.4% Malaysia; for old adults, 73.8% UK, 26.2% USA.

3 A minimum score of 13 was required to pass the test, and the test allowed for a maximum of 15 points for young adults and 18 points for older adults. Participants scored as follow: mean score young = 14.7, SD = +/- 0.7; mean score older = 16.8, SD = +/- 1.5.

4 On a screen of resolution of a 1920 × 1080, visual stimuli had a height of approximatively 1.5 cm and were displayed at around 4 cm distance of the fixation cross. However, please note that the relative size and distance depended on the participants’ screen size.

5 Because of the online configuration of this experiment, we used the two first blocks of single tasks as an attentional check, with the threshold of 70% level of incorrect responses in single tasks.

6 Also depending on auditory and visual trials.
